# Effects of transcranial electrical stimulation of the cerebellum, parietal cortex, anterior cingulate, and motor cortex on postural adaptation

**DOI:** 10.1038/s41598-025-92617-1

**Published:** 2025-04-08

**Authors:** Nastaran Bahadorani, Roya Khanmohammadi

**Affiliations:** 1 Physical Therapy Department, Rehabilitation Faculty, Tehran University of Medical Sciences, Tehran, Iran; 2 Physical Therapy Department, Rehabilitation Faculty, Tehran University of Medical Sciences, Tehran, Iran

**Keywords:** Transcranial direct current stimulation, Achilles tendon vibration, Center of pressure, Standing, Rehabilitation, Orthopaedics

## Abstract

Several cortical regions, such as the cerebellum, posterior parietal cortex (PPC), anterior cingulate cortex (ACC), and primary motor cortex (M1), play critical roles in postural adaptation. However, studies examining the effects of transcranial direct current stimulation (tDCS) on postural adaptation in healthy individuals are limited and often yield inconsistent findings, making it challenging to draw definitive conclusions. Most research has focused on individual brain regions, leaving a gap in understanding how the cerebellum, PPC, ACC, and M1 differentially contribute to postural adaptation. Identifying the most effective brain regions for postural adaptation could optimize rehabilitation strategies for individuals with postural control impairments. Thus, this study compared the effects of tDCS over these specific brain regions on postural adaptation. This parallel, randomized, double-blinded, controlled trial involved 75 participants, divided into five groups: anodal stimulation of the PPC, cerebellum, M1, ACC, or a sham group. Each group received 20 min of direct current stimulation in a single session. Center of pressure (COP) displacement, path length, velocity, and standard deviation (SD) were measured across three trials in the anteroposterior (AP) direction during standing disturbed using vibrators attached to bilateral Achilles tendons. A repeated measure ANOVA was used to assess within-group effects, while one-way ANOVA compared between-group differences. Between-group analysis did not reveal statistically significant differences during both the vibration and post-vibration phases. Nonetheless, the within-group analysis revealed significant enhancements in postural adaptation for the PPC and cerebellum groups during the vibration phase. Specifically, the PPC group demonstrated significant reductions in COP displacement (*P* = 0.005), path length (*P* = 0.018), and SD of COP displacement (*P* = 0.045) across trials. Similarly, in the cerebellar group, significant improvements were noted in COP displacement (*P* = 0.044), velocity (*P* = 0.006), and phase plane (*P* = 0.016) across trials. In contrast, no significant changes were found in the M1, ACC, or sham groups during either the vibration or post-vibration phases. In conclusion, while intergroup comparisons were not significant, intra-group analysis revealed that PPC and cerebellar stimulation significantly enhanced postural adaptation. Incorporating tDCS over the PPC or cerebellum in postural training programs could improve postural control, potentially reducing fall risk in clinical populations such as older adults or individuals with neurological dysfunction.

**RCT registration**: On the Iranian Registry of Clinical Trials (IRCT20220819055745N1). Registration date: 15/11/2022.

## Introduction

Maintaining balance and postural stability is essential for the safe and effective execution of daily tasks. The human postural control system integrates sensory inputs from the visual, vestibular, and somatosensory systems, which are processed by various regions of the central nervous system (CNS)^1^. Daily life involves numerous predictable and unpredictable disturbances that can threaten postural control, requiring continuous adaptation to ensure stability and prevent falls^2^.

Postural (motor) adaptation involves rapid adjustments to the postural control system, relying on sensory feedback to address disturbances^3^. This process generally occurs in real-time, requiring prompt modifications to sustain balance. Conversely, postural (motor) learning encompasses long-term changes in the brain and neuromuscular system, achieved through repetitive practice and ongoing feedback designed to enhance skill acquisition and performance progressively^4^. Consequently, adaptation is a quicker process than learning, focusing on altering existing behaviors rather than forming new ones in response to environmental or bodily changes. It is essential to acknowledge that these adaptations also provide the foundation for learning, eventually facilitating the acquisition of new skills^3,5^.

Consequently, postural adaptation is essential for human behavior and rehabilitation, allowing the nervous system to effectively handle changes in activities and adjust learned movement patterns to various situations^3^. This adaptation is vital for navigating environmental challenges and maintaining balance, significantly lowering the risk of falls, especially among older adults and individuals with balance issues^5^. Furthermore, effective postural control enables individuals to carry out daily activities safely, fostering functional independence. In rehabilitation contexts, postural adaptation is crucial for recovery from musculoskeletal and neurological injuries.

While the musculoskeletal and sensory systems play a significant role in postural control, the importance of the brain in processing and adapting to disturbances is becoming increasingly recognized^6^. Therefore, given the significance of the brain, recent advancements in neurorehabilitation have suggested that non-invasive brain stimulation techniques, like transcranial direct current stimulation (tDCS), which enhance cortical excitability, may improve postural control^7^.

Several cortical regions, including the cerebellum, parietal cortex, anterior cingulate cortex (ACC), and primary motor cortex (M1), have been associated with postural adaptation. Each of these areas has a specific function in integrating sensory information, detecting errors, and generating motor outputs required to adjust posture in response to disturbances^8–10^. However, there have been few studies examining the effectiveness of tDCS on postural adaptation in healthy individuals. For example, Young’s study found that bilateral stimulation of the posterior parietal cortex (PPC) (with one hemisphere receiving the cathode and the opposite hemisphere the anode electrode) resulted in weaker postural adaptation during a bipedal incline task compared to the sham group^11^. In contrast, Poortvliet’s research demonstrated that anodal cerebellar stimulation improved postural adaptation during an Achilles tendon vibration task in healthy participants^12^.

Several studies have also investigated the effectiveness of tDCS on motor adaptation^13–16^. For instance, Doppelmayr et al. examined the effects of stimulating the M1, cerebellum, and parietal cortex on motor adaptation during a mirror-drawing task, comparing these to a sham group. Their findings revealed that cerebellar anodal stimulation had the most significant impact on facilitating adaptation^13^. Additionally, M1 stimulation showed only a slight tendency to enhance performance during the initial phase, while parietal stimulation had no effect on adaptation^13^. Panico et al. demonstrated that simultaneous anodal stimulation over both the cerebellum and parietal regions improved motor adaptation^14^. Moreover, Jalali et al. explored the effects of tDCS on visuomotor adaptation across various task parameters, initially replicating previous findings of enhanced adaptation with cerebellar tDCS. However, they noted inconsistencies across different task parameters and a lack of reproducibility in a new participant group^15^. Additionally, Nettekoven et al. found that cerebellar anodal tDCS does not facilitate visuomotor adaptation^16^.

Overall, the studies are limited, and their results are sometimes contradictory, making it challenging to draw clear conclusions regarding the effectiveness of brain stimulation in clinical settings. Moreover, comprehensive studies comparing the effectiveness of stimulating different brain regions involved in postural adaptation are lacking. Most existing research has focused on individual regions, leaving a gap in understanding how the cerebellum, parietal cortex, anterior cingulate cortex, and primary motor cortex differentially contribute to postural adaptation. Understanding which brain regions contribute most critically to postural adaptation could optimize rehabilitation strategies for individuals with postural control impairments.

The PPC plays a vital role in the postural response to disturbances, largely due to its connections with motor and pre-motor areas and its function in integrating sensory-motor information^17,18^. Research involving stroke patients with damage to this area has shown that the PPC is active during reactive postural control in response to lateral disturbances^19^. Another important area is the ACC, which is essential for monitoring movement and detecting errors, particularly in the context of balance disturbances^20^. Its connections with various brain regions, such as the motor and pre-motor areas, facilitates effective coordination of motor outputs needed for posture adjustment^21^. Moreover, the cerebellum contributes to postural adaptation by fine-tuning motor programs^12^. It assists in motor control by predicting sensory input, constructing internal models, and adjusting them based on new sensory feedback^8^. The cerebellum, containing 80% of the brain’s neurons, has a complex network for inter-neuronal communication^22^. It forms reciprocal connections with several cortical areas, such as the primary motor cortex, pre-motor cortex, prefrontal cortex, temporal lobe, and posterior parietal cortex. These connections indicate that the cerebellum is involved in both motor and non-motor functions, influencing various behaviors through its interactions with other brain regions^22^. Another key area is the M1, which produces precise motor adjustments that are crucial for maintaining postural stability^10^. When postural disturbances arise, the M1 modulates motor output by increasing corticospinal excitability to enable proper motor responses. Moreover, M1 regulates postural muscles that contribute to stabilizing the trunk during balance-related tasks^23^.

It is evident that several brain regions contribute to postural adaptation, but determining which one has the greatest impact remains unclear. This study hypothesized that although stimulating all these regions could be advantageous, the cerebellum might prove to be the most effective due to its extensive network of connections, highlighting its involvement in a variety of functions.

One of the key methodological strengths of this study is the use of high-definition tDCS (HD-tDCS). Unlike conventional tDCS, which utilizes larger electrodes that produce more diffuse electric fields across the cortical surface, HD-tDCS provides more localized stimulation, allowing for greater precision in targeting specific brain regions. This enhances the ability to isolate and interpret the functions of targeted areas^24^. Finite element method (FEM) models indicate that HD-tDCS generates distinct electric field strength and distribution compared to conventional tDCS, reducing the uncontrolled spread of the electric field^25,26^. This results in improved spatial accuracy for stimulating specific cortical areas. Moreover, physiological studies suggest that HD-tDCS offers superior precision in modulating neurophysiological components^24,27^. Notably, one study demonstrated that HD-tDCS produces more enduring effects compared to conventional tDCS^27^.

## Method

### Study design

This study was a parallel, randomized, double-blinded, and controlled trial. Participants were randomly assigned to one of five groups: anodal stimulation of PPC, cerebellum, M1, ACC, or sham. All groups received the interventions in a laboratory setting. This study was registered as a clinical trial on the Iranian Registry of Clinical Trials (IRCT20220819055745N1) on 15/11/2022.

### Participants

75 healthy subjects were recruited from the university community through posters and social media advertisements (a simple non-probability sampling method)(Table 1). All participants provided written informed consent, and the study was approved by the Tehran University of Medical Sciences Ethics Committee (IR.TUMS.FNM.REC.1401.066(.

**Table 1:** Demographic characteristics and COP parameters during the 60-second baseline phase before brain stimulation across groups.

Parameters	PPC		CB		M1		ACC		Sham		*P* value
	Mean	SD	Mean	SD	Mean	SD	Mean	SD	Mean	SD	
Female (n (%))	6 (40)		9 (60)		7 (47)		10 (67)		7 (47)		0.577
Age (years)	21.73	3.22	22.07	2.22	21.21	3.24	22.13	3.46	21.33	1.59	0.841
Height (cm)	172.27	7.93	168.80	6.76	173.36	9.40	173.13	9.93	171.93	10.93	0.662
Weight (kg)	68.20	10.80	69.73	11.19	65.57	10.99	70.07	16.91	66.87	15.14	0.901
BMI (kg/m^2^)	22.87	2.46	24.39	2.98	21.72	2.60	23.10	3.27	22.71	3.42	0.207
COP displacement (cm)	2.74	0.76	2.46	0.80	3.18	2.72	2.51	0.90	2.84	0.99	0.627
COP path length (cm)	33.06	9.68	30.47	8.79	31.06	16.80	30.69	15.24	30.49	8.45	0.935
SD of COP displacement (cm)	0.34	0.12	0.32	0.10	0.36	0.23	0.31	0.13	0.34	0.14	0.808
Velocity of COP displacement (cm/s)	0.64	0.18	0.62	0.17	0.66	0.29	0.55	0.08	0.60	0.12	0.442
Phase plane	1.03	0.41	0.82	0.32	1.09	0.62	0.94	0.55	1.18	0.63	0.293

COP: center of pressure; SD: standard deviation; PPC: posterior parietal cortex; CB: cerebellum; M1: primary motor cortex; ACC: anterior cingulate cortex.

*P < 0.05 is significant.

The inclusion criteria were as follows: aged 18 to 40 years; no psychological or neurological disorders; no history of migraines; no history of epilepsy or seizure; no history of head injury resulting in a loss of consciousness; not having metallic implants, including surgical clips, intracranial electrodes, or a pacemaker; not being on prescription medication, or self-medicating^28^; no orthopedic, or rheumatological injuries; no intense physical activity within 2 h before testing.

### Outcome measures

The outcome measures included center of pressure (COP) displacement, COP path length, standard deviation (SD) of COP displacement, velocity of COP displacement, and phase plane in the anterior-posterior (AP) direction.

### Sample size

The sample size was determined using G*Power 3.1.9.2 software^29^ based on a pilot study, focusing on the COP displacement and velocity during vibration phase. The calculation used a within-between interaction effect size of 0.052 and 0.055, a power of 0.95, α = 0.05, non-sphericity correction (ε) = 1, and a correlation among repeated measures of 0.5. The analysis indicated that at least 75 participants were needed to detect a within-between interaction effect in a test design of 5 groups and 3 measurements with the specified parameters.

### Randomization and blinding

The participants were randomly assigned to one of five groups: anodal stimulation of PPC, cerebellum, M1, ACC, or sham with a 1:1 allocation ratio. Randomization was conducted using a web-based randomization service (http://www.randomization.com) with block randomization (block size = 10) managed by an independent third party. Details of the allocated groups were written on cards and concealed using sequentially numbered, opaque, sealed envelopes. In this way, the allocation sequence was concealed from the main researchers. In this study, in addition to participants, the investigator collecting the outcome measures was blinded to the group allocation.

### Procedure

In this study, a force plate (Bertec Corporation, Columbus, OH, USA) with a sampling frequency of 200 Hz was utilized. Measurements were taken across four phases: baseline before brain stimulation, baseline after brain stimulation, vibration phase, and post-vibration phase.

In phase of baseline before brain stimulation, participants were instructed to stand barefoot on the force plate for 60 s, maintaining a heel distance of 6 cm, a foot angle of 30 degrees, with their eyes close and arms at their sides to collect baseline data. The foot positions were marked on a footprint to ensure consistency for all participants during the experiment.

**Fig. 1:**
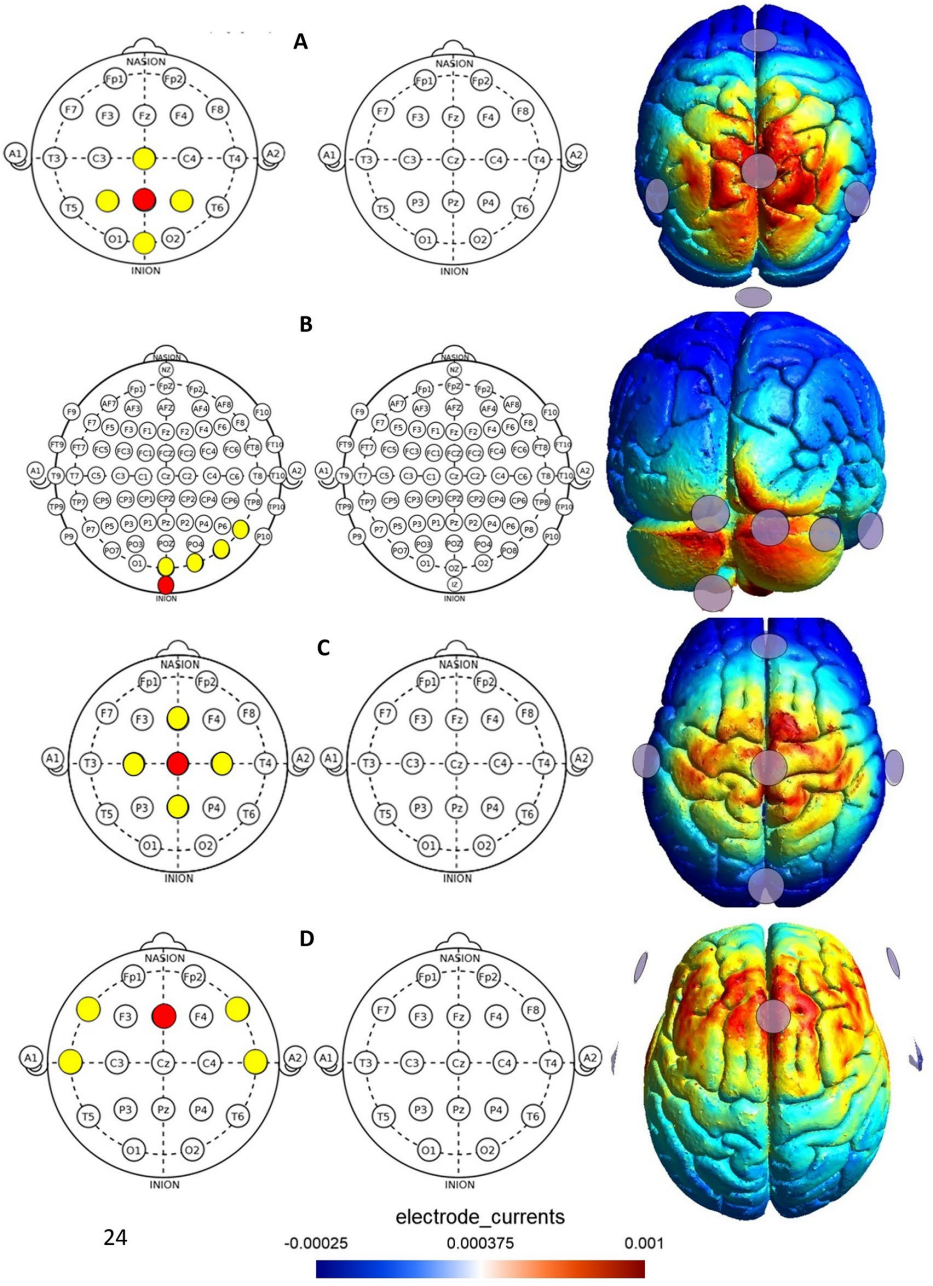
The electric field distributions for different montages (posterior parietal cortex (PPC), cerebellum, primary motor cortex (M1), and anterior cingulate cortex (ACC)). The electric field distributions have been simulated by open-source SimNIBS software. (**A**) For the PPC, the anode was placed at Pz of the 10/20 EEG template, and the other four electrodes were set at Cz, P3, P4, and Oz. (**B**) For the cerebellum, the anodal electrode was positioned over Iz, while the cathodal electrodes were placed on P8, O2, Oz, and PO8. (**C**) For M1, the anode was placed at Cz, and the other four return electrodes were placed over Fz, C3, C4, and Pz. (**D**) For the ACC, the anodal electrode was positioned on Fz, and cathodal electrodes were placed on T3, T4, F7, and F8. Warmer colors represent areas with higher electric field normal components, while cooler colors signify lower components.

After collecting the baseline data, participants underwent 20 min of stimulation using a Starstim tDCS device (Starstim, Neuroelectrics, Barcelona, Spain). They sat in a chair during this time. A 4 × 1 Ag/AgCl ring electrode montage (one anode electrode and four cathode electrodes) was used. The size of the electrodes was 3.14 cm, which were filled with conductive electrolyte gel. A realistic head model, including the scalp, skull, cerebrospinal fluid, and brain, was used for the finite element analysis. Direct curren^2^t was delivered for 20 min with an intensity of 1 mA (current density of 0.318 mA/cm^2^) in the anodal stimulation electrode and an intensity of − 0.25 mA (current density of -0.08 mA/cm^2^) in each cathode electrode. The first and last 10 s of the current were gradually ramped up (fade-in) or down (fade-out) to minimize discomfort. For the PPC region, the anodal electrode was positioned on Pz, with four cathodal electrodes on Cz, P3, P4, and Oz based on the 10/20 EEG template^30^. For the cerebellum, the anodal electrode was positioned over Iz, situated below Oz at 10% of the distance between the inion and nasion, while the cathodal electrodes were placed on P8, O2, Oz, and PO8^31^. For the M1, the anodal electrode was on Cz, with cathodal electrodes on C4, C3, Fz, and Pz^32^. For the ACC, the anodal electrode was positioned on Fz, and cathodal reference electrodes were placed on T3, T4, F7, and F8^33^. For sham stimulation, one of the electrode positions on the PPC, cerebellum, M1 or ACC was randomly selected, and the current was turned off after a 10-second fade-in period. The finite element model analysis in the SimNIBS software was utilized to optimize the placement of electrodes and the current parameters employed in the study. SimNIBS calculates the electric field distribution based on the specified stimulation parameters through computational modeling on a standard head model from the Montreal Neurological Institute (MNI), which includes the scalp, skull, cerebrospinal fluid, and brain. In this model, warmer colors signify areas with a higher electric field, whereas cooler colors denote regions with lower electric field components. Figure 1 illustrates the electric field distributions for different electrode configurations.

Following this, participants stood straight and barefoot, with their eyes closed and arms at their sides, on the force plate at the marked positions, where custom-made vibrating DC motors with eccentric weights (frequency 70–80 Hz, amplitude 1 mm) were secured to the bilateral Achilles tendon (above the calcaneal tuberosity at the level of the lateral malleoli) using straps. To prevent participants from anticipating the start of stimulation, the vibrators were activated 5 to 8 s after the baseline recording, with the vibration lasting for 15 s. After the vibration ceased, participants remained on the force plate for 25 s. This procedure was repeated for three trials, with a 1-minute rest between each trial. Consequently, four phases were recorded: a 60-second baseline phase before brain stimulation, a 5–8 s baseline phase after brain stimulation, a 15-second vibration phase, and a 25-second post-vibration phase^12^. This method was selected based on Poortvliet et al. research that examined the cerebellum’s role in postural adaptation, allowing us to compare the findings with at least one similar study.

Vibration stimulates the Ia afferents of muscle spindles, generating false sensory feedback about the current muscle length. This results in the perception of muscle elongation. A common response to this misperception is a backward bending to compensate for the perceived lengthening of the muscles. This immediate effect on body orientation can lead to increased fluctuations in COP, particularly when visual acuity is involved. The vibration in the Achilles tendon introduces a mismatch between the sensory information obtained from lower limb muscle stretch receptors and other sources, such as the vestibular system and joint or skin receptors, ultimately compromising postural stability^12,34^.

Postural stability is affected during both the application and removal of the vibration. When vibration is introduced, it distorts and conflicts with certain afferent signals, resulting in balance destabilization. Conversely, the removal of the vibration can also disrupt the perceived standing position, leading to further instability. The post- vibration instability is likely attributed, at least in part, to a temporary decrease in the discharge and sensitivity of muscle spindles, as well as the time required for the CNS to recalibrate the weighting of the existing sensory inputs^34^.

### Data analysis

For data analysis, the raw data was subjected to low-pass filtering with a zero-phase shift using a 6th-order Butterworth filter and a cutoff frequency of 10 Hz, utilizing MATLAB. In this study, only AP axis was analyzed. This focus was chosen because when the vibrator is attached to the back of the ankle joints, individuals tend to respond to the vibration by leaning backward, leading to increased fluctuations in the COP primarily in the anterior-posterior direction. Therefore, the primary objective was to investigate postural adaptations along this specific plane. The data from the vibration and post-vibration phases were extracted from the first five seconds before the onset of vibration (baseline phase after brain stimulation). The outcomes were calculated using the following equations.

$$\:COP\:Displacement\:\left(cm\right)=COP\:max-COP\:min$$

$$\:COP\:Path\:Length\:\left(cm\right)=\:{\sum\:}_{i=1}^{n-1}\sqrt{{\left({COP}_{i+1}-\:{COP}_{i}\right)}^{2}}$$

$$\:SD\:of\:COP\:Displacement\:\left(cm\right)=\:\sqrt{\frac{{\sum\:}_{i=1}^{n}{\left({COP}_{i\:}-\:\stackrel{-}{COP}\right)}^{2}}{n-1}}$$

$$\:Velocity\:of\:COP\:Displacement\:\left(\frac{cm}{s}\right)=\:\frac{COP\:Displacement}{\:t}$$

$$\:Phase\:Plane=\:\sqrt{{{\sigma\:}^{2}}_{COP\:Displacement}+\:{{\sigma\:}^{2}}_{velocity\:of\:COP\:Displacement}}$$

### Statistical analysis

Data were analyzed using SPSS version 23. Normality was assessed using the Shapiro-Wilk test, which confirmed that the data were normally distributed. Quantitative data were summarized using means and standard deviations, while qualitative variables were described using frequencies.

To compare demographic variables between groups, one-way ANOVA was used for quantitative variables, and the Chi-square test was employed for qualitative variables. Additionally, a one-way ANOVA was conducted to confirm the comparability of COP parameters during the 60-second baseline phase before brain stimulation across the groups.

A two-way mixed ANOVA was initially conducted to assess the main effects of group, trial, and their interaction in evaluating the intervention’s effects. When group-trial interactions were not significant, despite variations in group behaviors across trials, additional analyses were performed separately for within-group and between-group factors. This approach was taken to address potential limitations, such as the small sample size and unequal variances between groups.

One-way repeated measures ANOVA was employed to analyze trial effects within each group, while between-group differences were assessed using one-way ANOVA. For between-group comparisons, changes in parameters (e.g., trial 2 minus trial 1, trial 3 minus trial 1, and trial 3 minus trial 2) were calculated and compared. Bonferroni post-hoc tests were applied for multiple comparisons when ANOVA results were significant.

Effect sizes were reported to complement the statistical significance. Partial eta squared (η2) was used for ANOVA results and interpreted as small (0.01–0.06), medium (0.06–0.14), or large (≥ 0.14). For pairwise comparisons, Cohen’s d was calculated and categorized as small (0.2–0.5), medium (0.5–0.8), large (0.8–1.3), or very large (≥ 1.3). A significance level of *p* < 0.05 was applied for all tests.

## Results

### Demographic characteristics and baseline phase before brain stimulation

Table 1 displays the demographic characteristics and COP parameters recorded during the 60-second baseline phase before brain stimulation for all groups. The ANOVA results revealed no significant differences between the groups in terms of demographic characteristics and COP parameters during the baseline phase, confirming that the groups were comparable in terms of postural stability (*P* > 0.2).

### Between-group effects

The results showed that during both the vibration and post-vibration phases, the changes in COP parameters between the groups—comparing changes in trial 2 to trial 1, trial 3 to trial 1, and trial 3 to trial 2—were not statistically significant (Table 2).

**Table 2:** Analysis of between-group effects in both phases of vibration and post-vibration.

Parameters	Vibration Phase			Post-vibration Phase		
	F	*P*	Cohen’s d	F	*P*	Cohen’s d
Δ COP displacement_T2 vs. T1_	0.905	0.466	0.049	0.463	0.763	0.026
Δ COP displacement_T3 vs. T2_	0.881	0.480	0.048	0.376	0.825	0.021
Δ COP displacement_T3 vs. T1_	1.048	0.389	0.056	0.544	0.704	0.030
Δ COP path length_T2 vs. T1_	0.531	0.714	0.029	1.064	0.381	0.057
Δ COP path length_T3 vs. T2_	1.306	0.276	0.069	0.532	0.712	0.030
Δ COP path length_T3 vs. T1_	1.664	0.168	0.087	1.238	0.303	0.066
Δ SD of COP displacement_T2 vs. T1_	0.588	0.672	0.033	1.295	0.280	0.069
Δ SD of COP displacement_T3 vs. T2_	0.624	0.647	0.034	0.658	0.624	0.036
Δ SD of COP displacement_T3 vs. T1_	0.405	0.805	0.023	0.703	0.593	0.039
Δ Velocity of COP displacement_T2 vs. T1_	1.143	0.343	0.061	0.752	0.560	0.041
Δ Velocity of COP displacement_T3 vs. T2_	0.502	0.734	0.028	0.112	0.978	0.006
Δ Velocity of COP displacement_T3 vs. T1_	1.161	0.336	0.062	0.915	0.460	0.050
Δ Phase plane_T2 vs. T1_	1.512	0.208	0.080	0.597	0.666	0.033
Δ Phase plane_T3 vs. T2_	1.546	0.198	0.081	0.490	0.743	0.027
Δ Phase plane_T3 vs. T1_	1.144	0.343	0.061	0.711	0.587	0.039

COP: Center of Pressure; SD: Standard Deviation. * *P* < 0.05 is significant.

### Within-group effects

#### The effects of PPC stimulation

A one-way repeated measures ANOVA revealed that during the vibration phase, COP displacement was statistically significant across trials (F(2,28) = 6.463, *P* = 0.005, η^2^ = 0.316). Post hoc tests indicated that in the PPC group, COP displacement significantly decreased in trial 3 compared to trial 1, with a mean difference (MD) of -1.273, 95% CI: -2.266 to -0.280, *P* = 0.011 (Table 3).

**Table 3:** Descriptive statistics of COP parameters across three trials during the vibration phase and analysis of within-group effects.

Groups	Parameters	Trial 1		Trial 2		Trial 3		F	P	η^2^
		Mean	SD	Mean	SD	Mean	SD			
PPC	COP displacement (cm)	9.47	2.56	8.68	1.79	8.20	1.42	6.463	0.005*	0.316
	COP path length (cm)	32.31	21.15	25.46	12.21	21.37	9.98	6.194	0.018*	0.307
	SD of COP displacement (cm)	7.40	0.57	7.23	0.38	7.21	0.38	3.473	0.045*	0.199
	Velocity of COP displacement (cm/s)	1.12	1.21	0.70	0.75	0.68	0.86	2.880	0.073	0.171
	Phase plane	1.07	1.27	0.57	0.67	0.76	1.18	1.933	0.164	0.121
CB	COP displacement (cm)	9.95	2.07	8.91	1.16	8.65	1.14	4.297	0.044*	0.235
	COP path length (cm)	27.19	9.17	23.65	5.96	23.95	7.62	1.876	0.189	0.118
	SD of COP displacement (cm)	7.49	0.32	7.35	0.27	7.28	0.26	2.767	0.080	0.165
	Velocity of COP displacement (cm/s)	0.94	1.09	0.43	0.57	0.25	0.79	6.080	0.006*	0.303
	Phase plane	0.99	1.34	0.39	0.54	0.16	0.91	5.776	0.016*	0.292
M1	COP displacement (cm)	9.48	1.33	9.30	1.63	9.05	0.98	0.626	0.542	0.043
	COP path length (cm)	26.49	8.14	23.05	6.42	23.02	5.09	2.394	0.131	0.146
	SD of COP displacement (cm)	7.53	0.32	7.44	0.41	7.38	0.24	1.024	0.363	0.068
	Velocity of COP displacement (cm/s)	0.82	0.87	0.45	0.29	0.45	0.53	2.538	0.118	0.153
	Phase plane	1.03	1.49	0.36	0.30	0.41	0.45	3.807	2.913	0.106
ACC	COP displacement (cm)	11.18	4.22	9.46	1.90	9.62	1.60	2.442	0.131	0.149
	COP path length (cm)	31.51	15.20	27.39	7.43	26.97	6.43	1.643	0.221	0.105
	SD of COP displacement (cm)	7.77	0.77	7.44	0.52	7.53	0.42	2.178	0.153	0.135
	Velocity of COP displacement (cm/s)	0.76	0.75	0.50	0.55	0.58	0.45	2.079	0.159	0.129
	Phase plane	0.80	0.76	0.43	0.44	0.50	0.31	3.356	0.070	0.193
Sham	COP displacement (cm)	9.72	2.14	9.19	2.02	9.58	2.60	0.694	0.508	0.047
	COP path length (cm)	26.74	11.47	25.33	10.20	25.33	15.70	0.284	0.755	0.020
	SD of COP displacement (cm)	7.50	0.37	7.39	0.46	7.44	0.43	0.847	0.440	0.057
	Velocity of COP displacement (cm/s)	0.52	0.49	0.53	0.62	0.41	0.48	0.323	0.727	0.023
	Phase plane	0.53	0.38	0.65	0.53	0.43	0.49	1.108	0.344	0.073

COP: Center of Pressure; SD: Standard Deviation; PPC: Posterior Parietal Cortex; CB: Cerebellum; M1: Primary Motor Cortex; ACC: Anterior Cingulate Cortex.

*P < 0.05 is significant.

Additionally, in the same group during the vibration phase, ANOVA showed that COP path length was also statistically significant across trials (F(2,28) = 6.194, *P* = 0.018, η^2^ = 0.307). Bonferroni post hoc tests revealed that the COP path length significantly decreased in trial 3 compared to trial 1, with a MD of -10.937, 95% CI: -21.992 to 0.119, *P* = 0.05 (Table 3).

Furthermore, for the PPC group during the vibration phase, ANOVA demonstrated that the SD of COP displacement was statistically significant across trials (F(2,28) = 3.473, *P* = 0.045, η^2^ = 0.199). However, post hoc tests did not reveal significant differences between trials. In the most favorable comparison, the SD of COP displacement decreased in trial 3 compared to trial 1, with a MD of -0.190, 95% CI: -0.393 to 0.014, *P* = 0.071 (Table 3).

The one-way repeated measures ANOVA did not show statistically significant differences across trials during the post-vibration phase for any of the parameters (Table 4).

**Table 4:** Descriptive statistics of COP parameters across three trials during the post-vibration phase and analysis of within-group effects.

Groups	Parameters	Trial 1		Trial 2		Trial 3		F	P	η^2^
		Mean	SD	Mean	SD	Mean	SD			
PPC	COP displacement (cm)	9.89	3.59	9.55	2.68	9.07	1.68	1.043	0.366	0.069
	COP path length (cm)	32.59	16.36	30.46	10.01	27.85	8.02	1.844	0.177	0.116
	SD of COP displacement (cm)	7.27	0.37	7.27	0.47	7.25	0.28	0.047	0.906	0.003
	Velocity of COP displacement (cm/s)	0.38	0.60	0.18	0.61	0.15	0.37	1.414	0.260	0.092
	Phase plane	0.80	0.99	0.65	1.28	0.49	0.62	1.447	0.252	0.094
CB	COP displacement (cm)	9.78	2.16	9.84	1.95	9.38	1.49	0.496	0.614	0.034
	COP path length (cm)	30.53	9.52	27.06	7.75	27.78	7.74	1.631	0.214	0.104
	SD of COP displacement (cm)	7.35	0.32	7.40	0.27	7.29	0.22	0.653	0.528	0.045
	Velocity of COP displacement (cm/s)	0.34	0.71	0.21	0.50	0.15	0.77	0.616	0.547	0.042
	Phase plane	0.60	1.36	0.58	0.84	0.32	1.04	0.687	0.511	0.047
M1	COP displacement (cm)	11.21	3.32	10.27	2.86	10.04	2.62	1.574	0.225	0.101
	COP path length (cm)	31.31	9.84	30.54	10.52	28.37	9.54	0.516	0.603	0.036
	SD of COP displacement (cm)	7.66	0.66	7.42	0.49	7.47	0.64	1.621	0.223	0.104
	Velocity of COP displacement (cm/s)	0.24	0.35	0.07	0.41	0.05	0.35	1.117	0.341	0.074
	Phase plane	0.38	0.43	0.15	0.42	0.22	0.41	1.547	0.231	0.099
ACC	COP displacement (cm)	10.28	2.36	9.95	2.28	10.15	1.89	0.357	0.703	0.025
	COP path length (cm)	29.68	7.64	29.48	6.46	29.00	6.20	0.135	0.874	0.010
	SD of COP displacement (cm)	7.46	0.44	7.37	0.38	7.44	0.38	0.390	0.681	0.027
	Velocity of COP displacement (cm/s)	0.32	0.38	0.16	0.49	0.24	0.27	1.245	0.303	0.082
	Phase plane	0.57	0.52	0.36	0.61	0.30	0.30	2.722	0.083	0.163
Sham	COP displacement (cm)	10.39	2.66	10.27	2.58	10.38	2.96	0.017	0.983	0.001
	COP path length (cm)	27.13	12.74	29.73	12.98	31.14	15.59	0.695	0.437	0.047
	SD of COP displacement (cm)	7.42	0.24	7.42	0.36	7.50	0.45	0.470	0.630	0.032
	Velocity of COP displacement (cm/s)	0.10	0.29	0.17	0.42	0.21	0.48	0.310	0.736	0.022
	Phase plane	0.36	0.43	0.44	0.58	0.44	0.69	0.142	0.869	0.010

COP: Center of Pressure; SD: Standard Deviation; PPC: Posterior Parietal Cortex; CB: Cerebellum; M1: Primary Motor Cortex; ACC: Anterior Cingulate Cortex.

*P < 0.05 is significant.

#### The effects of cerebellar stimulation

A one-way repeated measures ANOVA revealed that in the cerebellar group during the vibration phase, COP displacement was statistically significant across trials (F(2,28) = 4.297, *P* = 0.044, η^2^ = 0.235). However, post hoc tests did not show significant differences between trials. The most notable reduction in COP displacement occurred in trial 2 compared to trial 1, with a MD of -1.035, 95% CI: -2.376 to 0.307, *P* = 0.164 (Table 3).

Additionally, in the same group during the vibration phase, ANOVA indicated that the velocity of COP displacement was statistically significant across trials (F(2,28) = 6.080, *P* = 0.006, η^2^ = 0.303). Bonferroni post hoc tests revealed that the velocity of COP displacement significantly decreased in trial 3 compared to trial 1 (MD: -0.692, 95% CI: -1.367 to -0.016, *P* = 0.044) and in trial 2 compared to trial 1 (MD: -0.518, 95% CI: -1.013 to -0.023, *P* = 0.039) (Table 3).

Furthermore, during the vibration phase, ANOVA demonstrated that the phase plane was statistically significant across trials (F(2,28) = 5.776, *P* = 0.016, η^2^ = 0.292). Post hoc tests showed that the phase plane significantly decreased in trial 3 relative to trial 1 (MD: -0.829, 95% CI: -1.684 to -0.026, *P* = 0.05) (Table 3).

The one-way repeated measures ANOVA did not show statistically significant differences across trials during the post-vibration phase for any of the parameters (Table 4).

#### The effects of M1 stimulation

The one-way repeated measures ANOVA did not reveal statistically significant differences across trials during either the vibration or post-vibration phases for any of the parameters (Tables 3 and 4).

#### The effects of ACC stimulation

The one-way repeated measures ANOVA did not indicate any statistically significant differences across trials during the vibration or post-vibration phases for any of the parameters (Tables 3 and 4).

#### The effects of Sham stimulation

The one-way repeated measures ANOVA did not show any statistically significant differences between trials in either the vibration or post-vibration phases for any of the parameters (Tables 3 and 4).

## Discussion

While intergroup comparisons did not reveal significant differences between the groups, intra-group analysis indicated that stimulation of the PPC and cerebellar regions significantly enhanced postural adaptation across trials. In contrast, no such effects were observed after stimulation of the M1, the ACC, or in the sham group.

The current study demonstrated that cerebellar stimulation enhances postural adaptability during the vibration phase. Previous research indicates that the cerebellum mediates compensatory postural reactions (feedback strategies) through sensory feedback loops while also contributing to predictive postural adjustments via feed-forward mechanisms^35^. Both feedback and feed-forward strategies are essential for maintaining postural stability. Thus, the observed increase in stability and postural adaptation during trials appears to be linked to the cerebellum’s role in enhancing these strategies. Additionally, the cerebellum is crucial for integrating various sensory information and converting that information into motor commands, establishing its anatomical connections with the parietal cortex and motor cortex^36^. One significant sensory system associated with the cerebellum is the vestibular system. The cerebellum integrates vestibular information with inputs from other sensory sources, such as vision and proprioception, to produce precise movements adjusted to the situation^37^. Moreover, the cerebellum plays a vital role in motor learning and adapting to environmental changes. This brain structure leverages prior experiences to enhance motor responses and adjust the weighting of vestibular information^38^. Consequently, it can be inferred that after applying vibration to the Achilles tendon on both sides while participants’ eyes were closed, the CNS compensates for the loss of proprioceptive feedback and visual input by relying on the vestibular system. By stimulating the cerebellum, this likely enhances the vestibulocerebellar pathways, thereby improving postural control.

The findings of this study are consistent with those of Poortvliet et al.,^12^ who found that both sham and anodal cerebellar stimulation improve postural adaptation, with the latter demonstrating significantly greater enhancements. This study shares methodological similarities with Poortvliet et al., particularly in administering stimulation before vibration to evaluate the aftereffects. Both studies employed tDCS at an intensity of 1 mA for 20 min, and vibration was applied to the Achilles tendon on both sides to create a disturbance. A key difference, however, is that the current study utilized HD-tDCS rather than conventional tDCS, allowing for more localized current distribution that targets the cerebellar region specifically. In contrast, conventional tDCS, which uses 7 × 5 carbon electrodes, may result in current dispersing to neighboring areas^12^.

Additionally, Doppelmayr et al. investigated the effects of stimulating the M1, cerebellum, and parietal cortex on motor adaptation during a mirror drawing task in comparison to a sham group. In that study, all stimulations were anodal and administered via HD-tDCS^13^. They found that anodal stimulation of the cerebellum had the greatest impact on promoting adaptation. In contrast, stimulation of the M1 only indicated a slight enhancement in adaptation during the initial phase, whereas stimulation of the parietal region had no effect on adaptation^13^. Concerning the cerebellum, the results of the current study are consistent with those of the Doppelmayr et al. study; however, the findings related to the parietal region do not exhibit the same level of consistency. The current study demonstrated that PPC stimulation enhances postural adaptability during the vibration phase.

Furthermore, these findings contrast with the results of Young et al., who employed an incline-intervention task to induce postural adaptation in healthy participants^11^. In their study, subjects first completed a 30-second baseline trial of quiet standing on a horizontal surface. They then stood on an inclined surface set at a 10° angle for 5 min before returning to the horizontal surface for another standing trial. The stimulation groups in this study comprised three conditions: (1) anodal stimulation of the right PPC combined with cathodal stimulation of the left PPC, (2) cathodal stimulation of the right PPC with anodal stimulation of the left PPC, and (3) a sham group. Conventional tDCS was used, with an electrode area of 25 cm^2^ and a stimulation intensity of 1.5 mA, involving all 15 subjects across all stimulation groups. The study concluded that asymmetric stimulation of the PPC—where one hemisphere was excitatory and the other inhibitory—reduced adaptation. They proposed that simultaneous activation of both PPCs (with both anodal stimulations) likely facilitates adaptation^11^. The present study employed symmetrical stimulation of the parietal cortex by positioning the anodal electrode at the Pz site, located at the midline of the head. This symmetrical approach could account for the positive effects of PPC stimulation on postural adaptation that we observed in our results.

Research indicates that the PPC is highly interconnected with several motor regions, including the M1 and the supplementary motor area (SMA)^39–41^. These connections support the integration of sensory information with motor commands essential for making postural adjustments^42^. Consequently, stimulating this area may improve sensorimotor integration, leading to more accurate posture adjustments. Additionally, the PPC integrates inputs from various sensory modalities, such as visual, auditory, and somatosensory systems. This multi-sensory integration is vital for spatial awareness and body orientation, which are necessary for adjusting posture^43^. Therefore, stimulating this region could enhance sensory feedback processing and improve the brain’s ability to map the body’s position in relation to its environment, resulting in better postural control and adaptation. Furthermore, the PPC is part of the fronto-parietal network that plays a role in spatial attention^44^. Functional MRI (fMRI) studies have demonstrated that heightened activation in the PPC is associated with an increase in attentional resources directed toward tasks that involve balance and motor control^45,46^. Thus, stimulation of this region may positively influence postural adaptation by enhancing the modulation of spatial attention related to balance and postural control.

Regarding M1 stimulation, the present study did not show significant changes across the three trials. A literature review revealed no studies examining the effect of M1 stimulation on postural adaptation in healthy young adults. However, one study investigated the effect of M1 stimulation on postural adaptation in individuals with Parkinson’s disease^47^. This study used a cross-over design with a two-week interval between sessions, examining the impact of different intensities of conventional tDCS (1 mA, 2 mA, and sham) for 20 min, with electrode areas of 35 cm^2^. Distribution was introduced using a moving support surface, and various parameters related to COP were recorded, including the time taken for COP variability to stabilize after perturbation, the onset of gastrocnemius muscle activity, and cortical activity in the prefrontal lobe. The results showed that both stimulation groups stabilized faster than the sham group, regardless of intensity. Additionally, prefrontal cortex activity on the stimulated side was significantly reduced, and muscle activity onset was quicker with 2 mA stimulation. However, no differences were observed across the seven trials, and contrary to the hypothesis, no reduction in the number of trials required for adaptation was seen. Like the present study, M1 stimulation did not improve postural adaptation. The authors suggested that this may be because postural responses improved from the first trial, leaving limited opportunity for further adaptation in subsequent trials, thus masking the effects of stimulation on adaptation^47^.

One key consideration when reviewing studies is distinguishing between those examining brain stimulation’s effects on motor (postural) learning versus those focused on motor (postural) adaptation. Learning and adaptation are related but distinct concepts in neuroscience and biomechanics^3,4^. As previously mentioned motor (postural) learning is a long-term process that improves through practice and repetition, leading to stable and lasting changes. In contrast, motor (postural) adaptation is a quicker process, enabling immediate responses to environmental changes. The primary goal of motor adaptation is to maintain balance and optimal performance in new or changing conditions^3,4^.

There is a perspective that M1 stimulation may be more effective for enhancing motor (postural) learning, while cerebellar stimulation is more effective for motor (postural) adaptation. Two relevant studies in this context are Kaminski et al. and Steiner et al.^48,49^. Kaminski et al.‘s study demonstrated that stimulating the M1 associated with leg muscles in a single session improved whole-body dynamic balance learning in healthy individuals. They used conventional tDCS with a 25 cm^2^ electrode for 20 min at an intensity of 1 mA^48^. Conversely, Steiner et al. used same task but applied cerebellar stimulation at 2 mA for 10 min, yielding no significant effects^49^. However, in the current study, which focused on postural adaptation, significant changes were observed in the cerebellum group during trials, while M1 stimulation did not produce similar results. Therefore, this may be explained by the interpretation that the motor cortex plays a more prominent role in postural learning, while the cerebellum has a greater impact on postural adaptation. However, further research is necessary to clarify this issue.

In the current study, brain stimulation was applied before vibration, allowing for an investigation of the stimulation’s after-effects. Research in motor learning suggests that the impact of tDCS on the M1 may be time-dependent^50–52^. This implies that applying tDCS during or prior to a task can yield different outcomes. Some studies indicate that M1 stimulation has positive effects when applied concurrently with a task (online)^50,51^. However, when used before a motor learning task (offline), it has been shown to either have no impact or even impair performance^50–52^. These findings have been attributed to homeostatic plasticity, which acts as a counterbalance to synaptic plasticity, preventing prolonged excessive excitability or depression to maintain neuronal activity within a certain range^53,54^. According to the Bienenstock-Cooper-Munro (BCM) theory, a postsynaptic activity threshold exists: below it, long-term depression (LTD) occurs, and above it, long-term potentiation (LTP) occurs. Anodal stimulation, which increases postsynaptic activity, raises this threshold, reducing the likelihood of LTP and increasing the chances of LTD. As a result, motor learning, which would typically induce LTP, might lead to LTD under these conditions, reducing learning and enhancing cortical inhibition^53^. Homeostatic plasticity counteracts the instabilities caused by synaptic plasticity^51^. However, regarding whether the effect M1 stimulation on postural adaptation is also similar to its effect on time-dependent motor learning, as far as we know, no study has been conducted. Therefore, further studies are needed to provide a more accurate answer in this area.

In this study, ACC stimulation also did not show a significant effect on postural adaptation. Overall, one potential reason for the lack of significant differences could be the high variance in the data, influenced by several factors. One factor is the small sample size, and another is the use of a parallel-group design instead of a crossover design. In parallel-group studies, different participants are compared, leading to higher variance compared to crossover studies. However, in postural learning and adaptation research, using parallel designs is often unavoidable because the possibility of one session being influenced by previous sessions is high, especially with multiple stimulation conditions.

Moreover, some studies have suggested that the variability in tDCS study results may be due to the inherent nature of tDCS, which has been shown to have low reproducibility and high result variance across studies. In this regard, Jalali et al. examined the reproducibility of cerebellar stimulation effects on a visual cursor rotation task, finding that repeating the experiment with a new group of subjects led to different outcomes^15^. Additionally, there is significant variability in the tasks used in existing studies, which contributes to diverse and sometimes uncertain results. Furthermore, most previous studies employed conventional tDCS, while this study used HD-tDCS. Conventional tDCS often leads to current spreading to non-target areas that may also be involved in postural control, potentially stimulating additional brain regions, which is a factor that should be considered when comparing results across studies. Another important consideration is that in this study, the control group was a sham group rather than a completely un-stimulated group. The sham group can still experience placebo effects due to the perceived stimulation. Placebo effects result from participants’ expectations and beliefs about the intervention’s efficacy. Some studies have suggested that tDCS may generate a strong placebo effect^55^. For instance, there is evidence that sham tDCS can increase cortical excitability^56^. Consequently, the absence of significant differences between stimulation groups and the control group, as well as the reduced effect size in between-group comparisons, may be attributed to the placebo effect in the control group.

## Study limitations

One limitation of this study is that postural adaptation was assessed only through biomechanical measures, without considering neurophysiological outcomes such as brain excitability. As a result, the underlying neurophysiological mechanisms of the brain stimulation effects remain unclear. Additionally, participants’ beliefs and expectations regarding the stimulation’s effects, which could influence performance, were not accounted for in the study. It is strongly recommended that future studies gather data on participants’ mental expectations through pre- and post-intervention questionnaires to evaluate their impact on the results. Furthermore, the study involved healthy young adults from an academic setting, limiting the generalizability of the findings to other populations.

## Conclusion

The notable intra-group improvements with PPC and cerebellum stimulation emphasize the crucial role these regions play in facilitating postural adaptation. In contrast, the lack of similar effects in the M1, ACC, and sham groups highlights the importance of precise targeting when aiming to enhance adaptive postural control. These results suggest that tDCS applied to the PPC and cerebellum could be a valuable tool for optimizing postural training in healthy individuals, offering a foundation for future exploration in broader populations. However, its potential benefits for clinical populations, such as older adults or those with neurological disorders remain uncertain and warrant further investigation. Future studies should determine whether these outcomes can be replicated in populations at greater risk of falls to inform evidence-based recommendations for neuromodulation therapies.

## Data Availability

Data are available upon reasonable request to the corresponding author via Email address.

## References

[1] Horak, F. B. & Macpherson, J. M. Postural orientation and equilibrium. *J. Compr. Physiol.*, 255–292 (1996).

[2] Edmunds, K. J. et al. Cortical recruitment and functional dynamics in postural control adaptation and habituation during vibratory proprioceptive stimulation. *J. Neural Eng.* **16**10.1088/1741-2552/ab0678 (2019). 10.1088/1741-2552/ab0678 30754028

[3] Bastian, A. J. Understanding sensorimotor adaptation and learning for rehabilitation. *Curr. Opin. Neurol.* **21**, 628–633. 10.1097/WCO.0b013e328315a293 (2008). 18989103 10.1097/WCO.0b013e328315a293 PMC2954436

[4] Leech, K. A., Roemmich, R. T., Gordon, J., Reisman, D. S. & Cherry-Allen, K. M. Updates in motor learning: implications for physical therapist practice and education. *Phys. Ther.* **102**10.1093/ptj/pzab250 (2022). 10.1093/ptj/pzab250 PMC8793168 34718787

[5] Wolpe, N. et al. Age-related reduction in motor adaptation: brain structural correlates and the role of explicit memory. *Neurobiol. Aging*. **90**, 13–23. 10.1016/j.neurobiolaging.2020.02.016 (2020). 32184030 10.1016/j.neurobiolaging.2020.02.016 PMC7181181

[6] Li, L., Zhang, S. & Dobson, J. The contribution of small and large sensory afferents to postural control in patients with peripheral neuropathy. *J. Sport Health Sci.* **8**, 218–227. 10.1016/j.jshs.2018.09.010 (2019). 31193300 10.1016/j.jshs.2018.09.010 PMC6523875

[7] Beretta, V. S. et al. Transcranial direct current stimulation for balance rehabilitation in neurological disorders: A systematic review and meta-analysis. *Ageing Res. Rev.* **81**, 101736. 10.1016/j.arr.2022.101736 (2022). 36116750 10.1016/j.arr.2022.101736

[8] Shadmehr, R. & Krakauer, J. W. A computational neuroanatomy for motor control. *Exp. Brain Res.* **185**, 359–381. 10.1007/s00221-008-1280-5 (2008). 18251019 10.1007/s00221-008-1280-5 PMC2553854

[9] Kelly, G. & Shanley, J. Rehabilitation of ataxic gait following cerebellar lesions: applying theory to practice. *Physiother. Theory Pract.* **32**, 430–437. 10.1080/09593985.2016.1202364 (2016). 27458875 10.1080/09593985.2016.1202364

[10] Whelan, P. J. The involvement of the motor cortex in postural control: a delicate balancing act. *J. Physiol.* **587**10.1113/jphysiol.2009.176750 (2009). 10.1113/jphysiol.2009.176750 PMC2746605 19648441

[11] Young, D. R., Parikh, P. J. & Layne, C. S. Non-invasive brain stimulation of the posterior parietal cortex alters postural adaptation. *Front. Hum. Neurosci.* **14**, 248. 10.3389/fnhum.2020.00248 (2020). 32676017 10.3389/fnhum.2020.00248 PMC7333640

[12] Poortvliet, P., Hsieh, B., Cresswell, A., Au, J. & Meinzer, M. Cerebellar transcranial direct current stimulation improves adaptive postural control. *Clin. Neurophysiol.* **129**, 33–41. 10.1016/j.clinph.2017.09.118 (2018). 29136550 10.1016/j.clinph.2017.09.118

[13] Doppelmayr, M., Pixa, N. H. & Steinberg, F. Cerebellar, but not motor or parietal, high-density anodal transcranial direct current stimulation facilitates motor adaptation. *J. Int. Neuropsychological Society: JINS*. **22**, 928–936. 10.1017/s1355617716000345 (2016). 10.1017/S1355617716000345 27152869

[14] Panico, F., Sagliano, L., Sorbino, G. & Trojano, L. Engagement of a parieto-cerebellar network in prism adaptation. A double-blind high-definition transcranial direct current stimulation study on healthy individuals. *Cortex; J. Devoted Study Nerv. Syst. Behav.* **146**, 39–49. 10.1016/j.cortex.2021.10.005 (2022). 10.1016/j.cortex.2021.10.005 34818617

[15] Jalali, R., Miall, R. C. & Galea, J. M. No consistent effect of cerebellar transcranial direct current stimulation on visuomotor adaptation. *J. Neurophysiol.* **118**, 655–665. 10.1152/jn.00896.2016 (2017). 28298304 10.1152/jn.00896.2016 PMC5539446

[16] Nettekoven, C. R., Jurdon, R., Nandi, T., Jenkinson, N. & Stagg, C. J. Cerebellar anodal tDCS does not facilitate visuomotor adaptation or retention. *Brain Stimul.* **15**, 1435–1438. 10.1016/j.brs.2022.10.006 (2022). 36341957 10.1016/j.brs.2022.10.006 PMC7613922

[17] Cattaneo, L., Giampiccolo, D., Meneghelli, P., Tramontano, V. & Sala, F. Cortico-cortical connectivity between the superior and inferior parietal lobules and the motor cortex assessed by intraoperative dual cortical stimulation. *Brain Stimul.* **13**, 819–831. 10.1016/j.brs.2020.02.023 (2020). 32289713 10.1016/j.brs.2020.02.023

[18] Wise, S. P., Boussaoud, D., Johnson, P. B. & Caminiti, R. Premotor and parietal cortex: corticocortical connectivity and combinatorial computations. *Annu. Rev. Neurosci.* **20**, 25–42. 10.1146/annurev.neuro.20.1.25 (1997). 9056706 10.1146/annurev.neuro.20.1.25

[19] Lin, Y. H. et al. Reactive postural control deficits in patients with posterior parietal cortex lesions after stroke and the influence of auditory cueing. *Am. J. Phys. Med. Rehabil.* **93**, 849–859. 10.1097/phm.0000000000000093 (2014). 24901758 10.1097/PHM.0000000000000093

[20] Orr, C. & Hester, R. Error-related anterior cingulate cortex activity and the prediction of conscious error awareness. *Front. Hum. Neurosci.* **6**, 177. 10.3389/fnhum.2012.00177 (2012). 22723775 10.3389/fnhum.2012.00177 PMC3377932

[21] Paus, T. Primate anterior cingulate cortex: where motor control, drive and cognition interface. *Nat. Rev. Neurosci.* **2**, 417–424. 10.1038/35077500 (2001). 11389475 10.1038/35077500

[22] Prati, J. M., Pontes-Silva, A. & Gianlorenço, A. C. L. The cerebellum and its connections to other brain structures involved in motor and non-motor functions: A comprehensive review. *Behav. Brain. Res.* **465**, 114933. 10.1016/j.bbr.2024.114933 (2024). 38458437 10.1016/j.bbr.2024.114933

[23] Nandi, T., Fisher, B. E., Hortobágyi, T. & Salem, G. J. Increasing mediolateral standing sway is associated with increasing corticospinal excitability, and decreasing M1 Inhibition and facilitation. *Gait Posture*. **60**, 135–140. 10.1016/j.gaitpost.2017.11.021 (2018). 29202358 10.1016/j.gaitpost.2017.11.021

[24] Edwards, D. et al. Physiological and modeling evidence for focal transcranial electrical brain stimulation in humans: a basis for high-definition tDCS. *NeuroImage* **74**, 266–275 10.1016/j.neuroimage.2013.01.042 (2013). 10.1016/j.neuroimage.2013.01.042 PMC4359173 23370061

[25] Datta, A. et al. Gyri-precise head model of transcranial direct current stimulation: improved Spatial focality using a ring electrode versus conventional rectangular pad. *Brain Stimul.* **2**, 201–207. 10.1016/j.brs.2009.03.005 (2009). 207.e201. 20648973 10.1016/j.brs.2009.03.005 PMC2790295

[26] Alam, M., Truong, D. Q., Khadka, N. & Bikson, M. Spatial and polarity precision of concentric high-definition transcranial direct current stimulation (HD-tDCS). *Phys. Med. Biol.* **61**, 4506–4521. 10.1088/0031-9155/61/12/4506 (2016). 27223853 10.1088/0031-9155/61/12/4506

[27] Kuo, H. I. et al. Comparing cortical plasticity induced by conventional and high-definition 4 × 1 ring tDCS: a neurophysiological study. *Brain Stimul.* **6**, 644–648. 10.1016/j.brs.2012.09.010 (2013). 23149292 10.1016/j.brs.2012.09.010

[28] Thair, H., Holloway, A. L., Newport, R. & Smith, A. D. Transcranial direct current stimulation (tDCS): A beginner’s guide for design and implementation. *Front. NeuroSci.* **11**, 641. 10.3389/fnins.2017.00641 (2017). 29213226 10.3389/fnins.2017.00641 PMC5702643

[29] Faul, F., Erdfelder, E., Lang, A. G. & Buchner, A. G*Power 3: a flexible statistical power analysis program for the social, behavioral, and biomedical sciences. *Behav. Res. Methods*. **39**, 175–191. 10.3758/bf03193146 (2007). 17695343 10.3758/bf03193146

[30] Guo, Y. et al. Effects of Long-Lasting High-Definition transcranial direct current stimulation in chronic disorders of consciousness: A pilot study. *Front. Neurosci.* **13**, 412. 10.3389/fnins.2019.00412 (2019). 10.3389/fnins.2019.00412 PMC6502996 31114475

[31] Zhang, X., Hancock, R. & Santaniello, S. Transcranial direct current stimulation of cerebellum alters spiking precision in cerebellar cortex: A modeling study of cellular responses. *PLoS Comput. Biol.* **17**, e1009609. 10.1371/journal.pcbi.1009609 (2021). 34882680 10.1371/journal.pcbi.1009609 PMC8691604

[32] Xiao, S., Wang, B., Zhang, X., Zhou, J. & Fu, W. Acute effects of high-definition transcranial direct current stimulation on foot muscle strength, passive ankle kinesthesia, and static balance: A pilot study. *Brain Sci.* **10**10.3390/brainsci10040246 (2020). 10.3390/brainsci10040246 PMC7226500 32326228

[33] Leong, S. L. et al. High definition transcranial Pink noise stimulation of anterior cingulate cortex on food craving: an explorative study. *Appetite* **120**, 673–678. 10.1016/j.appet.2017.10.034 (2018). 29079475 10.1016/j.appet.2017.10.034

[34] Dettmer, M., Pourmoghaddam, A., O’Connor, D. P. & Layne, C. S. Interaction of support surface stability and Achilles tendon vibration during a postural adaptation task. *Hum. Mov. Sci.* **32**, 214–227. 10.1016/j.humov.2012.12.002 (2013). 23465726 10.1016/j.humov.2012.12.002

[35] Marchese, S. M., Farinelli, V., Bolzoni, F., Esposti, R. & Cavallari, P. Overview of the cerebellar function in anticipatory postural adjustments and of the compensatory mechanisms developing in neural dysfunctions. *J. Appl. Sci.* **10**, 5088 (2020).

[36] Rondi-Reig, L., Paradis, A. L., Lefort, J. M., Babayan, B. M. & Tobin, C. How the cerebellum May monitor sensory information for spatial representation. *Front. Syst. Neurosci.* **8**, 205. 10.3389/fnsys.2014.00205 (2014). 25408638 10.3389/fnsys.2014.00205 PMC4219422

[37] Angelaki, D. E. & Cullen, K. E. Vestibular system: the many facets of a multimodal sense. *Annu. Rev. Neurosci.* **31**, 125–150. 10.1146/annurev.neuro.31.060407.125555 (2008). 18338968 10.1146/annurev.neuro.31.060407.125555

[38] Bastian, A. J. Learning to predict the future: the cerebellum adapts feedforward movement control. *Curr. Opin. Neurobiol.* **16**, 645–649. 10.1016/j.conb.2006.08.016 (2006). 17071073 10.1016/j.conb.2006.08.016

[39] Chao, C. C. et al. Induction of motor associative plasticity in the posterior parietal cortex-primary motor network. *Cereb. Cortex* **25**, 365–373 10.1093/cercor/bht230 (2015). 10.1093/cercor/bht230 PMC4303801 23968834

[40] Breveglieri, R. et al. Functional connectivity at rest between the human medial posterior parietal cortex and the primary motor cortex detected by Paired-Pulse transcranial magnetic stimulation. *Brain Sci.* **11**10.3390/brainsci11101357 (2021). 10.3390/brainsci11101357 PMC8534070 34679421

[41] Gharbawie, O. A., Stepniewska, I. & Kaas, J. H. Cortical connections of functional zones in posterior parietal cortex and frontal cortex motor regions in new world monkeys. *Cereb. Cortex*. **21**, 1981–2002. 10.1093/cercor/bhq260 (2011). 21263034 10.1093/cercor/bhq260 PMC3155600

[42] Graziano, M. The organization of behavioral repertoire in motor cortex. *Annu. Rev. Neurosci.* **29**, 105–134. 10.1146/annurev.neuro.29.051605.112924 (2006). 16776581 10.1146/annurev.neuro.29.051605.112924

[43] Colby, C. L. & Goldberg, M. E. Space and attention in parietal cortex. *Annu. Rev. Neurosci.* **22**, 319–349. 10.1146/annurev.neuro.22.1.319 (1999). 10202542 10.1146/annurev.neuro.22.1.319

[44] Corbetta, M. & Shulman, G. L. Control of goal-directed and stimulus-driven attention in the brain. *Nat. Rev. Neurosci.* **3**, 201–215. 10.1038/nrn755 (2002). 11994752 10.1038/nrn755

[45] Greenberg, A. S., Esterman, M., Wilson, D., Serences, J. T. & Yantis, S. Control of spatial and feature-based attention in frontoparietal cortex. *J. Neuroscience: Official J. Soc. Neurosci.* **30**, 14330–14339. 10.1523/jneurosci.4248-09.2010 (2010). 10.1523/JNEUROSCI.4248-09.2010 PMC3307052 20980588

[46] Gitelman, D. R. et al. A large-scale distributed network for covert spatial attention: further anatomical delineation based on stringent behavioural and cognitive controls. *Brain: J. Neurol.* **122** (Pt 6), 1093–1106. 10.1093/brain/122.6.1093 (1999). 10.1093/brain/122.6.1093 10356062

[47] Beretta, V. S. et al. Effect of different intensities of transcranial direct current stimulation on postural response to external perturbation in patients with Parkinson’s disease. *Neurorehabilit. Neural Repair*. **34**, 1009–1019. 10.1177/1545968320962513 (2020). 10.1177/1545968320962513 33000679

[48] Kaminski, E. et al. Transcranial direct current stimulation (tDCS) over primary motor cortex leg area promotes dynamic balance task performance. *Clin. Neurophysiol.* **127**, 2455–2462. 10.1016/j.clinph.2016.03.018 (2016). 27178865 10.1016/j.clinph.2016.03.018

[49] Steiner, K. M. et al. Cerebellar tDCS does not improve learning in a complex whole body dynamic balance task in young healthy subjects. *PloS One*. **11**, e0163598. 10.1371/journal.pone.0163598 (2016). 27669151 10.1371/journal.pone.0163598 PMC5036893

[50] Sriraman, A., Oishi, T. & Madhavan, S. Timing-dependent priming effects of tDCS on ankle motor skill learning. *Brain Res.* **1581**, 23–29. 10.1016/j.brainres.2014.07.021 (2014). 25063361 10.1016/j.brainres.2014.07.021 PMC4166556

[51] Stagg, C. J. et al. Polarity and timing-dependent effects of transcranial direct current stimulation in explicit motor learning. *Neuropsychologia* **49**, 800–804. 10.1016/j.neuropsychologia.2011.02.009 (2011). 21335013 10.1016/j.neuropsychologia.2011.02.009 PMC3083512

[52] Rezaei, S. & Khanmohammadi, R. Comparison of short- and long-term effects of neurofeedback and transcranial electrical stimulation on the motor learning in healthy adults. *Behav. Brain. Res.* **476**, 115263. 10.1016/j.bbr.2024.115263 (2024). 39307285 10.1016/j.bbr.2024.115263

[53] Amadi, U., Allman, C., Johansen-Berg, H. & Stagg, C. J. The homeostatic interaction between anodal transcranial direct current stimulation and motor learning in humans is related to GABAA activity. *Brain Stimul.* **8**, 898–905. 10.1016/j.brs.2015.04.010 (2015). 26279408 10.1016/j.brs.2015.04.010 PMC4742653

[54] Karabanov, A. et al. Consensus paper: probing homeostatic plasticity of human cortex with Non-invasive transcranial brain stimulation. *Brain Stimul.* **8**, 442–454. 10.1016/j.brs.2015.01.404 (2015). 26050599 10.1016/j.brs.2015.01.404

[55] Braga, M. et al. The role of expectation and beliefs on the effects of non-Invasive brain stimulation. *Brain Sci.* **11**10.3390/brainsci11111526 (2021). 10.3390/brainsci11111526 PMC8615662 34827526

[56] Horvath, J. C., Vogrin, S. J., Carter, O., Cook, M. J. & Forte, J. D. Effects of a common transcranial direct current stimulation (tDCS) protocol on motor evoked potentials found to be highly variable within individuals over 9 testing sessions. *Exp. Brain Res.* **234**, 2629–2642. 10.1007/s00221-016-4667-8 (2016). 27150317 10.1007/s00221-016-4667-8

